# On the Management and Treatment of Hæmoptysis

**Published:** 1908-05-16

**Authors:** William MacLennan

**Affiliations:** Assistant Physician, Western Infirmary, Glasgow; Lecturer, Materia Medica and Therapeutics, Queen Margaret College, Glasgow University; Clinical Assistant to the Regius Professor of Medicine, Glasgow University


### Hospital Clinics.

### ON THE MANAGEMENT AND TREATMENT OF HÆMOPTYSIS.

By WILLIAM MacLENNAN, M.B., Assistant Physician, Western Infirmary, Glasgow; Lecturer, Materia Medica and Therapeutics, Queen Margaret College, Glasgow University; Clinical Assistant to the Regius Professor of Medicine, Glasgow University.

It must be the experience of most physicians that no form of sudden illness is associated with greater alarm to a patient than an attack of severe hæmoptysis. These hæmorrhages, when profuse, are the cause of much anxiety to the practitioner called on to deal with them. Fortunately, in the majority of instances a fatal termination is infrequent; but as death may result from syncope, all our energies should be exerted to combat rapidly and efficiently these grave accidents and to prevent the serious debility or shorten the long convalescence which are so common after a copious hæmorrhage. Hæmorrhages of a slight character may be left to take their normal course, for, if they are not oft repeated, they do little harm and may even act the part of safety valves and relieve local congestions.

Hæmoptysis and hæmatemesis must necessarily, on many occasions, be liable to confusion. The first duty of the physician is to differentiate the two conditions, as rapidly as may be, by a review of the whole circumstances and history of the case. It is only by making a correct diagnosis of the source of the hæmorrhage, and by correctly appreciating whether it comes from a large vessel or from the capillaries, that the physician can initiate any successful treatment.

Hæmoptysis is, perhaps, more common in pulmonary tuberculosis than in any other intrathoracic lesion. It is often the first and the earliest sign of that disease, and when profuse its treatment requires all the skill that the practitioner can bring to bear on it. In phthisis pulmonalis it is due to the erosion of a smaller or larger vessel, or to the rupture of a small aneurysm into a cavity of the lung. The amount of external hæmorrhage depends principally on two factors: (1) the size of the vessel ruptured, and (2) the accessibility of the blood to a bronchus. It thus happens that, clinically, we have hæmoptysis varying in degree from the slightest tinging or streaking of the sputum to a copious stream of almost pure blood. In the former case we are almost always dealing with an oozing or leak from the capillaries, or an overflow from a "pulmonary apoplexy," while in the latter we are concerned with the rupture of a vessel of considerable size. Hæmoptysis due to cardiac disease is usually gradual in its onset, and the blood is rarely liberated from the lung in a rush. Mitral stenosis is, however, occasionally associated with a severe hæmoptysis; and in pulmonary infarction a sharp bleeding is rather the rule than the exception.

There are certain essentials common to the treatment of the "slight" and "great" pulmonary hæmorrhages.

The decubitus of the patient is not a matter of indifference in the major hæmorrhages, although it may be of little moment in those which are slight. Almost by instinct, in most acute thoracic conditions the patient assumes the position which is most favourable to promote his comfort; but in the case of a severe hæmoptysis he requires some guidance. The site of the lesion should, when possible, be ascertained and the patient's position arranged accordingly. With the shoulders well propped up, he should be placed on the side from which the bleeding is coming. This tends greatly to minimise regurgitation into the sound lung, and so to lessen the risk of asphyxia. The possibility of subsequent broncho-pneumonia and dissemination of the tubercle bacilli is also thereby diminished. When an aneurysm of some size bursts into a cavity in communication with a bronchus, the flow of blood may be so profuse as to flood the sound lung and necessitate the employment of artificial respiration and the procedure for resuscitating the apparently drowned.

All fussy interference should be avoided and the patient's excitement soothed by encouragement. But the harrassing cough often defeats our efforts to attain rest: if this be violent and incessant it must be quieted. The straining causes a considerable rise in the blood-pressure and is a new source of danger. Except in those cases where actual asphyxia, from the volume of blood regurgitated, is imminent, the more we can do to quiet the cough while the hæmorrhage is still active, the less likely is any fatal syncope. By far the best remedy to administer in such an emergency is opium, and the hypodermic injection of morphine answers all the requirements. When a sedative action has thus quickly been attained it may be kept up by small doses of codeine or heroin. If morphine be given in doses greater than are required to exert a controlling effect only, it may do more harm than good. By suppressing the cough it causes the retention of blood, which tends to disseminate itself and produce broncho-pneumonia or new foci of tubercular infection.

In hæmoptysis the diet ought to be light and restricted. Large doses of fluid are contra-indicated. I am opposed to the common procedure of giving all fluids iced, or of permitting the patient to suck ice freely. One has only to reflect on the *rôle* that the abdominal vessels and circulation play, as regulators of the general blood-pressure, to be convinced of this. If it be kept in mind that the abdominal circulation is more under the influence of vaso-motor action than that of any other in the body, and indeed may largely dominate the general tension of the circulation, it is evident that large quantities of ice or iced drinks by contracting these vessels will raise the blood-pressure in the lungs and elsewhere and bring about the very effect we most desire to avoid. On this assumption I have never permitted the use of ice or iced drinks till the active stage of a severe hæmorrhage is over, and I am satisfied, from numerous observations, that hot drinks and warm compresses to the abdomen are far more efficacious. These, by dilating the abdominal vessels, reduce blood-pressure and so diminish a pulmonary hæmorrhage.

If it be probable that a bleeding is coming from a superficial part of the lung, a compress of ice applied directly over it may, by causing local vaso-constriction, co-operate with the abdominal dilatation. If the site of the lesion cannot be ascertained, no attempt should be made to apply cold locally.

Large volumes of fluid, which after a hæmorrhage are absorbed with great rapidity, ought never to be given, as they raise the pressure and diminish the coagulability of the blood. For the same reason the routine use of dilute saline cathartics is harmful, and the rectal or subcutaneous injection of a pint or more of fluid is to be condemned. The latter should only be used when the volume of blood is so reduced that a fatal syncope seems imminent. If a saline cathartic is to be given with the object of reducing the blood-pressure, it ought to be given concentrated, so as to attract water to itself from the intestinal mucous membrane. To-day we can obtain, by the exhibition of various other therapeutic agents, a more rapid, certain, and sustained decrease in pressure.

Routine treatment has held the field far too long. The attempt to treat all pulmonary hæmorrhages in the same way is unscientific and should be condemned as dangerous. For clinical purposes we must draw a sharp distinction between "slight" and "severe" hæmorrhages. These two varieties of bleeding, arising as they do respectively from capillaries and from vessels of greater magnitude, require important modifications in their management. If for these two conditions we attempt to use the same therapeutic measures we may convert a small into a large bleeding or increase the severity of a formidable one.

Nature spontaneously arrests a bleeding in two ways: (1) By depletion of the circulation so that the tension is reduced even to the point of bringing the patient to the verge of syncope; (2) by the coagulation of the blood at the point of rupture. In many instances these two methods, doubtless, cooperate with each other. In hæmorrhage from the rupture of a large vessel, the first is the usual cause of the spontaneous cessation of the bleeding; while in that from a smaller, it is the latter method that plays the predominating part. In dealing, therefore, with hæmoptysis we must be guided by the steps initiated by nature and aid her efforts to prevent a serious or fatal loss of blood. We have at our disposal certain drugs that enable us to bring about one or other of the conditions favourable to the arrest of the hæmorrhage.

Too much confidence has been placed in those remedies belonging to the "tannic acid" and "mineral astringent" groups, which have little or no action on remote hæmorrhages. Undoubtedly, they have a powerful hæmostatic effect, when they can act locally, by coagulating the blood. But they are not absorbed as such, and their decomposition products which circulate in the blood do not coagulate albumen. As a group, these preparations may be discarded as useless in pulmonary and other internal hæmorrhages.

But we have in calcium chloride a remedy which can be relied upon with a degree of certainty to promote blood-coagulation. For small capillary hæmorrhages calcium chloride alone may be sufficient: for a very small thrombus is required to seal these vessels. It may also be used in the major bleedings in conjunction with other remedies that act in a different fashion. This drug is best given in doses of from 15 to 20 grains dissolved in some syrup of lemon or of tolu to disguise its somewhat disagreeable taste. In the smaller hæmorrhages of pulmonary phthisis, hæmorrhagic pneumonia, and pulmonary infarction we have no better remedy. If there is any obstacle to the oral administration of the drug, it may be given, in a larger dose, through a long catheter per rectum. Or it may be employed subcutaneously, if a neutral preparation be used, in doses of from 3 to 4 grains. In bleedings that require prompt measures the somewhat slow action following the oral or rectal administration of this drug indicates its use rather by hypodermic injection. If calcium chloride is to do any good, it does so without delay, and its prolonged use is unnecessary: for the effect of the drug is only temporary.

Inhalations of turpentine are sometimes of service, and the administration of large quantities of gelatin tend to favour clotting of the blood. Gelatin acts most promptly and efficiently when given as an intravenous injection; but so employed, unless thoroughly sterilised, there is the risk of introducing with it pathogenic organisms into the blood.

Even more error is made in the routine use of ergot than in the employment of the ordinary astringents. The effects produced by ergot must be carefully considered in relation to the kind of hæmorrhage we are treating. The employment of this drug may be fraught with real danger for all except the bleedings from the smaller vessels. In common with digitalis it contracts the arterioles and increases the force of cardiac action. Clearly the action of ergot, in increasing the obstruction in front and the force of the pump behind, must be greatly to increase the tension of the blood in the intermediate larger vessels and thus aggravate seriously a hæmorrhage coming from one of these. In a few such cases I have seen a bleeding, that was showing signs of abating, promptly become active after a hypodermic injection of ergot. The very reason that contra-indicates the use of ergot, in a profuse arterial hæmorrhage coming from a larger vessel, makes it an ideal remedy for the treatment of an ooze from the rupture of some smaller vessels. Here the very vessels that are involved are deprived of their blood supply by vaso-constrictor action, and a hæmostatic action is brought about.

Experiment has proved that suprarenal material, when injected into the blood-vessels has a very rapid effect in raising the blood-pressure, and for the time being greatly strengthens the heart's action. Its effect is therefore not dissimilar in these respects to that of digitalis. But as the drug is quickly destroyed in the circulation or in the tissues, its action is of short duration. Although pharmacologists are well aware of the fact that the hypodermic or oral administration of adrenalin produces no general contraction of the peripheral vessels and no increase of cardiac force, yet physicians continue to prescribe this remedy through these channels for all kinds of hæmorrhages and for various conditions, such as exophthalmic goître and Addison's disease, where there is vaso-motor paralysis. Because the drug has a striking action in staunching bleeding from small vessels when applied locally, owing to its powerful vaso-constricting properties, it does not follow that it will have the same action remotely. But even though the drug were introduced directly into the veins, and the then certain general vaso-constrictor action obtained, it would only be suitable for capillary oozings and would be contra-indicated as dangerous in the larger hæmorrhages, just as ergot and digitalis are.

While, therefore, adrenalin is not clinically available as a remote hæmostatic, it may be of great value if employed intravenously in supporting the heart under certain conditions. Prior to the administration of an anæsthetic in debilitated subjects, or in operations on certain conditions specially associated with a liability to syncope, such as removal of the thyroid or thymus, the intravenous injection of a small dose of adrenalin would greatly minimise the risk of cardiac failure.

If hæmorrhage is recurrent and associated with a high tension of the circulation, one of the nitrites may be employed. In cardiac hæmoptysis due to an engorgement of the pulmonary vessels, especially if associated with much lividity or other signs of passive hyperæmia, the older physicians resorted to blood-letting. But such cases can be dealt with as successfully by an initial dose of amyl nitrite given by inhalation, followed later on by a member of the group which has a more sustained action, such as trinitrin. In the severer forms of hæmoptysis of pulmonary disease an inhalation of a full dose of amyl nitrite is the best remedy we possess. Instead of the external bleeding practised by the older physicians, this remedy produces a "bleeding" into the peripheral vessels throughout the entire body with a fall in blood-pressure that depletes the larger vessels and so arrests a sharp pulmonary hæmorrhage. Subsequent doses of trinitrin will maintain the reduction and permit of stasis and coagulation in the ruptured vessel. It is obvious, therefore, that the action of amyl nitrite would increase a capillary bleeding, and that its use must be restricted to the hæmorrhages from the larger arteries.

While these other medicinal remedies are being administered, bandages applied firmly round each upper limb, as near the trunk as possible, will arrest the venous return and produce a relative pulmonary anæmia. These bandages may be kept on for some minutes, and then very gradually removed so as to avoid any sudden increase of tension in the pulmonary arteries.

When a bleeding threatens to continue in spite of all these measures, or if we are dealing with a case of so-called "hæmorrhagic phthisis" with recurrent attacks of severe hæmoptysis, direct pulmonary compression ought to be tried. In two such cases I found it very efficient. Pulmonary compression is easy of production. Through a fine cannula introduced into the pleural cavity a sufficient quantity of oxygen gas is passed to compress and diminish slightly the movements of the lung. Oxygen is absorbed readily, but the compression is long enough exerted to permit of the formation of a coagulum in the ruptured vessel. If a more lasting compression be desired, or necessary, nitrogen gas is substituted for oxygen. Not being readily absorbed, it remains *in situ*, and the prolonged pressure permits of complete healing. The attendant risks of such a compression must, of course, be accepted.

